# Long-term Effectiveness of Patient-Directed Education to Sustain Deprescribing: A Hybrid Implementation Trial

**DOI:** 10.1093/geroni/igaf122.1650

**Published:** 2025-12-31

**Authors:** Amy Linsky, Katie Jones, Kelly Stolzmann, Jacquelyn Pendergast, Christopher Miller, Michael Still, Amy Rosen, Jolie Wormwood

**Affiliations:** VA Boston Health Care System, Boston, Massachusetts, United States; VA Boston Health Care System, Boston, Massachusetts, United States; VA Boston Health Care System, Boston, Massachusetts, United States; VA Boston Health Care System, Boston, Massachusetts, United States; VA Boston Health Care System, Boston, Massachusetts, United States; VA Boston Health Care System, Boston, Massachusetts, United States; VA Boston Health Care System, Boston, Massachusetts, United States; University of New Hampshire, Durham, New Hampshire, United States

## Abstract

Deprescribing, stopping or dose-reducing medication, can avoid harms from potentially inappropriate medications (PIMs). We examined the effectiveness, sustainability, and safety of a patient-engagement strategy in a hybrid effectiveness-implementation pragmatic trial at three Veterans Affairs facilities. Subjects were mailed brochures for one of three PIMs (proton pump inhibitors, high-dose gabapentin, diabetes agents with hypoglycemia risk) if they had chronic active prescriptions before a primary care provider (PCP) visit. Control subjects received usual care. We determined PIM deprescribing (cessation or any dose reduction) 6- and 12-months after the visit, categorizing participants into four patterns. A multinomial mixed effect logistic regression with patients nested in PCPs, adjusting for intervention cohort, PIM, and site, tested the intervention effect on patterns. We assessed for possible adverse drug withdrawal events (ADWEs). Compared to controls (n = 2,448), intervention patients (n = 2,480) were significantly older (M = 70.8 vs. M = 70.0, p = 0.012) and less likely to be White (82% vs. 83%, p = 0.017). Intervention patients had more sustained deprescribing (6 and 12 months: 19.3% vs 15.1%), but comparable short-term (6 months only: 9.8% vs 10.5%) and delayed (12 months only: 13.9% vs 14.5%) deprescribing. In the adjusted regression, intervention patients were significantly more likely to exhibit sustained deprescribing (OR = 1.32 [95%CI=1.13, 1.55]) but not short-term (OR = 0.96 [95%CI=0.79, 1.16]) or delayed (OR = 0.99 [95%CI=0.84, 1.17]) deprescribing. There were five potential ADWEs (0.2% incidence rate); three assessed as not related and two as possibly related to the intervention. Despite the low intensity, patient-directed education materials are an effective implementation strategy to promote and sustain deprescribing with minimal ADWEs.

